# Caveolin-1 in the regulation of cell metabolism: a cancer perspective

**DOI:** 10.1186/s12943-016-0558-7

**Published:** 2016-11-16

**Authors:** Zeribe Chike Nwosu, Matthias Philip Ebert, Steven Dooley, Christoph Meyer

**Affiliations:** 1 Department of Medicine II, Medical Faculty Mannheim, University of Heidelberg, Mannheim, 68167 Germany; 2 Molecular Hepatology Section, Medical Faculty Mannheim, University of Heidelberg, Mannheim, 68167 Germany

**Keywords:** CAV1, Glycolysis, Glutaminolysis, Mitochondrion, Fatty acid metabolism, Autophagy, Metabolic targets

## Abstract

Caveolin-1 (CAV1) is an oncogenic membrane protein associated with endocytosis, extracellular matrix organisation, cholesterol distribution, cell migration and signaling. Recent studies reveal that CAV1 is involved in metabolic alterations – a critical strategy adopted by cancer cells to their survival advantage. Consequently, research findings suggest that CAV1, which is altered in several cancer types, influences tumour development or progression by controlling metabolism. Understanding the molecular interplay between CAV1 and metabolism could help uncover druggable metabolic targets or pathways of clinical relevance in cancer therapy. Here we review from a cancer perspective, the findings that CAV1 modulates cell metabolism with a focus on glycolysis, mitochondrial bioenergetics, glutaminolysis, fatty acid metabolism, and autophagy.

## Background

Cancer cells have anomalous metabolic features, which enable them to adapt to constraints in the microenvironment [1, 2]. Otto Warburg proposed that cancer cells have impaired mitochondrial function and as such rely on high turnover of a rather inefficient aerobic glycolysis instead of oxidative phosphorylation (OXPHOS) [3–5]. While aerobic glycolysis (often called Warburg effect) largely holds true in several tumours, it is now known that tumours also retain normal mitochondrial function, using glutaminolysis to support proliferation via tricarboxylic acid (TCA) cycle [5, 6]. New evidences further highlight the importance of lipid metabolism, the serine pathway, autophagic alanine secretion, and macropinocytosis-mediated use of extracellular proteins for sustaining cancer nutrition and growth activities [5, 7–11]. Metabolic alterations in tumours are coordinated by several genes, prominent among which are *TP53*, *MYC* oncogene and hypoxia inducible factor 1α (*HIF1A*) [8, 9, 12–16]. In addition, metabolic enzymes such as pyruvate kinase muscle isoform (PKM2), lactate dehydrogenase A (LDHA), phosphofructose kinase A (PFKA) isoform, isocitrate dehydrogenase (IDH) and phosphoglycerate kinase (PGK1) confer oncogenic phenotypes when altered [17]. Furthermore, metabolites notably called “oncometabolites” (e.g. hydroxyglutarate, fumarate, lactate and ketones) can promote tumourigenesis in diverse ways, including by increasing cancer stemness, upregulating oncogene expression and reactive oxygen species amplification [18–22]. A detailed insight on molecular regulators of metabolic alterations could enhance the prospects of identifying cancer drug targets. In this article, we review evidences that CAV1 – a protein known to regulate cholesterol distribution, signal transduction, cell migration, and endocytic vesicular trafficking [23–25] – represents one such regulator of cell metabolism, especially in cancer cells.

## Relevance of Caveolin-1 in cancer

Caveolin-1 is a 22 kDa protein encoded by *CAV1* gene, and occupies flask-shaped plasma membrane invaginations called caveolae. It is one of three known caveolins (CAV1, 2 and 3) and is ubiquitously expressed in all cell types as is CAV2; CAV3 is mostly found in skeletal muscles [23, 25]. Besides earlier studies that implicated CAV1 in endocytosis, signaling and lipid disorders, research activities in the last two decades also focused on clarifying its relevance in cancer [23–33]. Consequently, CAV1 was found to be overexpressed in cancers of liver, colon, breast, kidney, lung, among others [29], and acts as a tumour promoter or suppressor depending on tumour type and stage [23, 33]. Regarding its tumour promoting function, it has been reported that high expression of CAV1 drives tumourigenesis by inhibiting apoptosis, facilitating anchorage-independent growth, drug resistance as well as metastasis [30, 33–39]. For instance, CAV1 expression in liver cancer patients was found to positively correlate with differentiation status, increased portal vein invasion, intrahepatic metastasis, and to predict overall survival outcome [37]. Accordingly, in vitro mechanistic study showed that CAV1 overexpression induced known mediators of migration and invasion, namely matrix metalloproteinases 2 and 9, and vascular epidermal growth factor [37]. Indeed, CAV1 and caveolae, which mediate molecular trafficking and contain signaling molecules such as non-receptor tyrosine kinases and endothelial nitric oxide synthase (eNOS), have long been proposed as potential therapeutic targets for disrupting tumour angiogenesis, progression and metastasis [40].

On the other hand, CAV1 acts as a tumour suppressor in some settings in that its low expression favours tumour progression [41–43]. For instance, in NIH3T3 cells oncogenically transformed by H-Ras induction, high CAV1 expression in the mitochondria reduced cell proliferation [43]. Codeficiency of CAV1 and the tumour suppressor, adenomatous polyposis coli, enhanced colorectal tumourigenesis in mice [44]. Furthermore, loss of stromal CAV1 in human breast cancer is associated with increased tumour recurrence, metastasis and poor clinical outcome [41]. Consistently, and contrary to its tumour promoting function highlighted above [37], high CAV1 expression improved overall survival in liver cancer patients, ostensibly by countering eNOS activity [42]. Altogether, accumulating evidences consistently support that CAV1 plays an important role in cancer progression – the specific nature of which seems to depend on several factors, including cancer type and stage, lesions on *CAV1* or its associated genes, its protein expression level and subcellular localisation. The fact that CAV1 could serve as a clinical biomarker [45, 46] further emphasizes its importance in cancer. However, despite knowledge of its expression pattern and roles in different cancers, it is still unclear whether CAV1 expression is a property that accompanies or directly drives altered metabolism, or if changes in energy balance modulate CAV1 level towards or against cancer progression.

## CAV1 in glycolysis

**Fig. 1:**
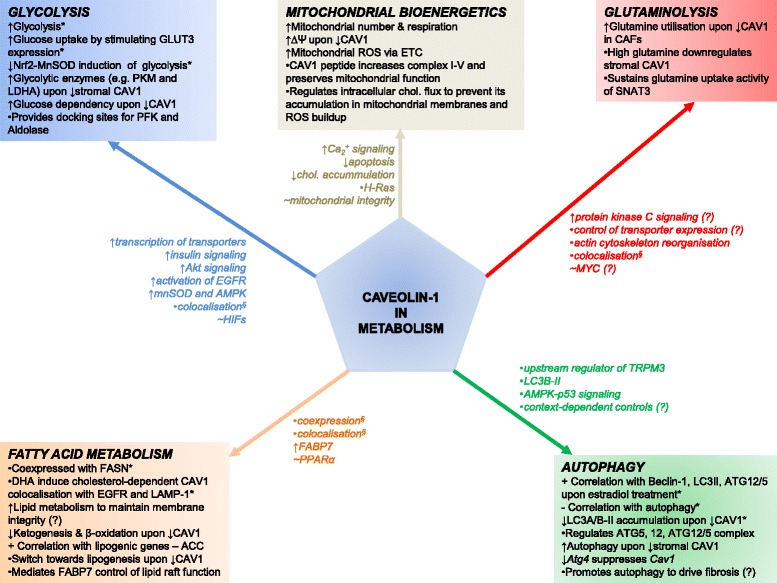
CAV1 influences metabolic processes in normal and cancer cells

**Fig. 2:**
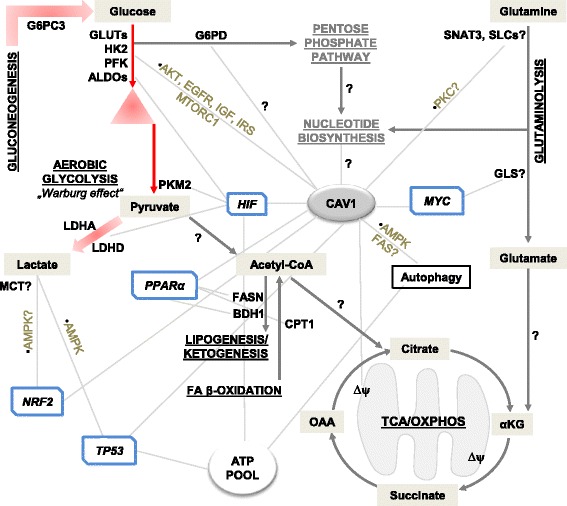
Schematic representation of metabolic processes and targets associated with CAV1 based on research findings

The preference of cancer cells for aerobic glycolysis is an evasive pro-survival strategy. This makes glycolysis an attractive therapeutic target in cancer, especially if its molecular regulators are identified and well characterized. Several studies reveal that CAV1 is involved in the modulation of glycolytic activities (Figs. 1 and 2). For instance, CAV1 expressing colon cancer cells undergo increased glycolysis upon exposure to inhalation anaesthesia (isoflurane), and are thus protected from tumour necrosis factor associated apoptosis [47]. High CAV1 expression in advanced colon cancer increased glucose uptake and ATP production by stimulating transcription of glucose transporter 3 (GLUT3, encoded by *SLC2A3*) [48]. Further, knockdown of CAV1 reduced cellular glucose uptake and lactate output (indicative of suppressed Warburg effect), reduced intracellular ATP, and triggered autophagy through activation of AMPK-p53 signaling [48]. In prostate cancer, immunoprecipitation and signaling studies showed that CAV1 interacts with insulin- and IGF-1 receptors (IR/IGF-1R), and can stimulate IR kinase activities by also interacting with low-density lipoprotein receptor-related protein 6 (LRP6). Accordingly, both LRP6 and CAV1 stimulate IR/IGF-1R signaling and, through AKT signaling activation, enhance glucose uptake, lactate output and cell proliferation – these effects correlated positively with hexokinase 2 (HK2) and GLUT3 expression [49]. Noteworthy, however, the authors reported that the expression of some glycolytic enzymes like enolase 1 (ENO1), PKM2 and LDHA were unchanged upon CAV1 knockdown.

Furthermore, CAV1 enhanced sensitivity to anti-diabetic drug (metformin) in a study comparing two non-small-cell lung cancer cell lines, Calu-1 and Calu-6, which express high and low CAV1 levels, respectively [50]. Specifically, metformin reduced phosphorylation of IGF-1 receptor substrates [AKT and Forkhead transcription factor 3a (FOXO3A)], suppressed IGF1-dependent cell proliferation, and increased AMPK phosphorylation as well as AMP/ATP ratio in Calu-1. In line, ectopic overexpression of CAV1 in Calu-6 enhanced sensitivity to metformin, as observed by increased AMP/ATP ratio and AMPK phosphorylation [50]. This CAV1-mediated metformin sensitivity may extend beyond glycolysis since the drug also acts on complex I of the electron transport chain in cancer cells; hence it offers further clue for investigating CAV1 expression and response to anti-metabolic drugs.

**Fig. 3:**
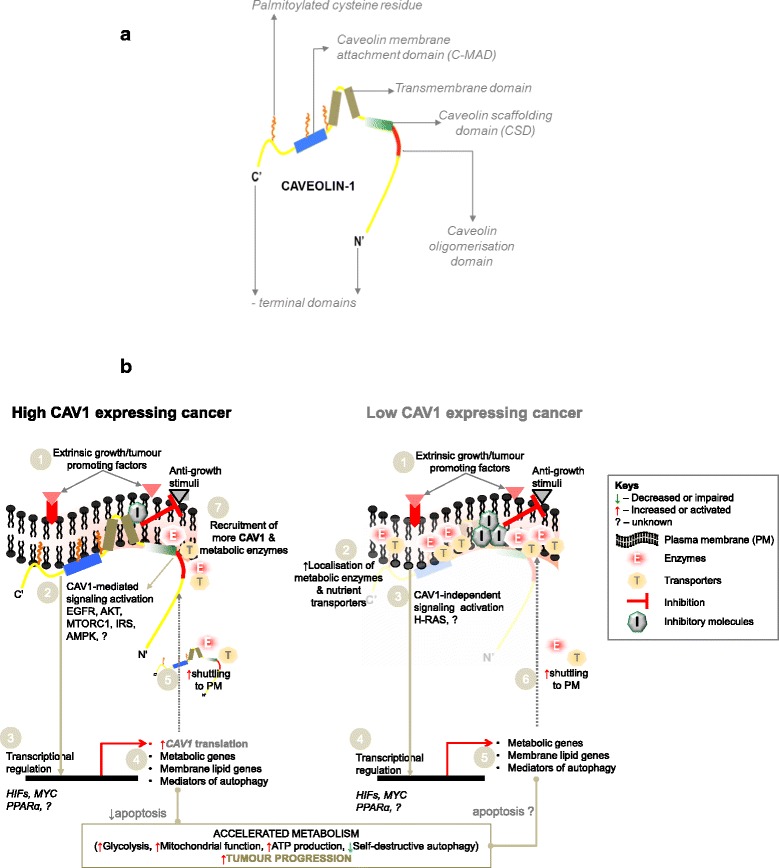
CAV1 and a hypothetical model of its role in cancer cell metabolism. a. A simplified diagram of CAV1. b. Model depicting CAV1 and its potential influence on metabolism in cancer cells expressing it in high or low levels

CAV1 provides a docking site for glycolytic enzymes. For instance, in caveolae of vascular smooth muscle cells and lymphocytes, the rate-limiting glycolytic enzyme phosphofructose kinase (PFK) as well as aldolase co-localised with CAV1 by binding to its scaffolding domain [51]. Likewise, the intracellular metabolite profile of endothelial cells revealed a decrease in glycolytic intermediates (e.g. 3-phosphoglycerate, fructose-6-phosphate and glucose-6-phosphate) upon CAV1 knockdown, indicating perturbed glycolysis, even though the effect on glucose consumption was not reported [52]. Thus, it highlights an interesting possibility that CAV1 could influence the localisation of relevant glycolytic mediators in cancer as depicted in Fig. 3b.

There are contexts in which CAV1 level is inversely related to glycolytic phenotypes. This has been reported in its crosstalk with HIFs, which are crucial transcriptional regulators of genes encoding glycolytic enzymes, glucose transporters, and also mediators of angiogenesis and tumour metastasis [53]. For instance, CAV1 is a direct target of HIF1 and HIF2, and is upregulated under hypoxia in clear cell renal cell carcinoma [54]. However, overexpression of activated HIF1α reduced CAV1 level, caused upregulation of glycolytic enzymes towards enhanced aerobic glycolysis, and increased lactate output in immortalized fibroblasts (hTERT-BJ1 cells). The fibroblasts expressing activated HIF1α also promoted xenograft tumour growth upon co-injection with breast cancer cells (MDA-MB-231) in nude mice [55]. This implies that downregulation of CAV1 in the stromal fibroblast compartment – in parallel with increased aerobic glycolysis – is a mechanism by which HIF1α promotes cancer progression. Furthermore, upregulation of several glycolytic enzymes, including aldolase A, enolase 1, PGK1, PKM2 and LDHA was observed in bone marrow derived stromal cells from *Cav1* knockout (KO) mice [41]. In invasive ductal carcinoma, CAV1 is reduced at the early stage of progression and predicts poor survival outcome. Mechanistically, reduced CAV1 expression enabled the induction of transcription factor NRF2 (NF-E2-related factor 2), which activates anti-oxidant manganese superoxide dismutase (MnSOD) that triggers AMPK-dependent glycolysis [56]. As a proof, ectopic expression of CAV1 in invasive ductal carcinoma cells (MCF7) suppressed NRF2 expression, the induction of MnSOD, and decreased aerobic glycolytic phenotype as measured by extracellular acidification and lactate output [56]. Further evidences have suggested that low CAV1 expression correlate with high reliance on glucose metabolism. For example, CAV1 deficiency increased carbohydrate metabolism in mice as determined by high respiratory exchange ratio, and prevented a metabolic switch to free fatty acids including during fasting. Specifically, hepatic gluconeogenesis emerged as a possible pathway to sustain glucose supply in *Cav1*-KO mice as revealed by upregulation of glucose-6-phosphatase (G6PC3) and LDHD [57]. Moreover, in a study on three different CAV1-deficient mouse strains, loss of CAV1 delayed liver regeneration and decreased survival after partial hepatectomy [58]. In the strain where regeneration still occurred, the loss of CAV1 was compensated by increased dependence on hepatic carbohydrate metabolism, as blocking glycolysis with glucose analogue (2-deoxy-glucose) further reduced survival and suppressed liver regeneration [58]. In a cancer context, low CAV1 and high glycolytic phenotypes would suggest a tumour suppressor function, but contradicts its role as a tumour promoter as discussed earlier. Taken together, the underlying mechanisms for the apparently context-dependent links between CAV1 and glycolysis remain largely unclear. More studies are required to elucidate the influence of CAV1 on glucose utilisation and glycolytic enzymes, especially in CAV1 overexpressing tumours, and to determine whether glycolysis modulates the expression and function of CAV1 in normal and cancer cells.

## CAV1 in mitochondrial bioenergetics

Beyond aerobic glycolysis, cancer cells still retain intact mitochondrial functions and can use alternative metabolites to support energy production. CAV1 has been associated with mitochondrial number and bioenergetic function in various cell types. For instance, mitochondria of hepatic cells in hypercholesterolemic rabbits were found to have high localisation of CAV1 [59]. Intravenous injection of mice with antennapedia-CAV1 (AP-CAV1) peptide–to increase CAV1 translocation across cell membrane–led to a darker electron-dense mitochondrial matrix, lower superoxide dismutase and catalase activity as compared to controls, suggesting that CAV1 is required for maintaining mitochondrial architecture and function. AP-CAV1 treatment also restored respiratory chain subunit proteins (complexes I–V), preserved mitochondrial function and suppressed apoptotic cell death [59]. Consistently, increased CAV1 expression was observed in an in vitro model of active microglia along with increased mitochondrial number, respiration, and glycolysis [60]. Colon cancer cells (HCT116) that overexpress CAV1 also had its abundant localisation in the mitochondria and, in the low expressing cells (HT29), overexpression of CAV1 led to its enrichment in the mitochondria and reduced apoptosis [61]. These findings provide clues that CAV1 could enhance mitochondrial functions to support tumour progression, details of which are yet to be fully understood. On the other hand, high CAV1 in the mitochondria suppresses proliferation in H-Ras driven tumour cell model [43]. Specifically, neoplastic transformation triggered by oncogenic H-Ras12v in NIH3T3 cells suppresses basal intracellular calcium (Ca^2+^) level, Ca^2+^ influx and also CAV1 expression. Further, reintroduction of CAV1 enhanced Ca^2+^ uptake into mitochondria, suppressed cell growth, colony formation, and induced apoptosis, implying that high CAV1 level may suppress mitochondrial function as a mechanism to mediate a suppressor activity in H-Ras-driven tumours [43].

CAV1 may modulate mitochondrial function by regulating cholesterol flux. In line with this notion, loss of CAV1 in mouse embryonic fibroblasts (MEFs) led to cholesterol accumulation in mitochondrial membrane, and increased reactive oxygen species (ROS) and cell death upon OXPHOS activation [62]. Increased circulating ROS was also observed in *Cav1*-KO mice, in which gonadal white adipose tissue presented with mitochondrial dysfunction. The increase in ROS was attributed to high mitochondrial membrane potential as further validated in vitro with CAV1-deficient MEFs [57]. However, the authors suggested that impaired mitochondrial function is a tissue-specific consequence of CAV1 loss, as comparison of oxygen consumption rates in liver and lung tissues revealed divergent results [57]. In cultured bovine aortic endothelial cells, knockdown of CAV1 caused an increase in mitochondrial ROS production, intracellular H_2_O_2_, and reduced the intracellular redox balance indicator [the ratio of reduced to oxidized glutathione (GSH/GSSG)] [52]. In the same study, *Cav1*-KO mice had increased oxidative stress as determined by high level of 8-isoprostane (a biomarker of lipid peroxidation and oxidative stress). Noteworthy, although membrane potential was increased, suppressed CAV1 expression did not significantly alter mitochondria abundance [52]. Altogether, these observations strongly link CAV1 expression to mitochondrial function, and could be relevant in understanding how CAV1 affects mitochondrial bioenergetics in cancer.

## Glutaminolysis – CAV1 in the alternative tumour energy pathway?

Glutaminolysis is a metabolic process in which glutamine is converted to α-ketoglutarate for onward utilisation in TCA cycle. Through this process, glutamine serves as alternative energy-rich substrate for cancer cells to meet the excessive nutritional demands that result from rapid proliferation [6, 63]. Currently, there is a limited insight on the association between CAV1 and glutaminolysis (Figs. 1 and 2). However, it has been proposed that glutamine availability inversely correlates with CAV1 expression in tumour stromal compartment, and that fibroblasts lacking CAV1 secrete more glutamine [64]. Accordingly, cancer associated fibroblasts cultured in high glutamine media had suppressed CAV1 expression and increased autophagy [64]. It is unclear how the observed loss of CAV1 and high glutamine availability work to drive tumour progression; however, the authors suggested that autophagy in the fibroblasts may serve as a key source of energy-rich glutamine to fuel mitochondrial activity in adjacent cancer cells [64]. This proposition aligns with inverse association of CAV1 with autophagy induction as reported by some studies (discussed later), but does not establish a direct link between enhanced glutaminolysis and high CAV1 expression as also seen in cancer.

Enhanced glutaminolysis via glutaminase induction is a known transcriptional function of *MYC* in cancer [14, 65]. Its encoded protein, c-MYC, regulates CAV1 and both promote tumour progression in prostate and colon cancer [49, 66–69]. Hence, although not yet proven, CAV1 could play a role in c-MYC-driven glutamine metabolism in tumours. It is also probable that CAV1 regulates the expression and localisation of amino acid transporters in plasma membrane. Consistent with the latter, the glutamine uptake activity of sodium neutral amino acid transporter 3 (SNAT3) was abolished following its coexpression with dominant-negative CAV1 mutant in *Xenopus laevis* oocytes [70]. The CAV1 mutant, when expressed 2 days after expressing SNAT3, prevented the rapid suppression of glutamine uptake by protein kinase C activator, phorbol-12-myristate-13-acetate [70]. These findings raise the possibility that CAV1 controls glutamine uptake and utilisation in cancer by influencing SNAT3 or other amino acid transporters reviewed in [71, 72], and could lead to novel understanding of glutamine dependency in cancer cells.

## CAV1 in fatty acid metabolism

Several studies have reported that CAV1 modulates fatty acid metabolism in normal and cancer cells (Fig. 1). Fatty acid synthase (FASN) is a crucial enzyme in fatty acid biosynthesis, and catalyzes the formation of palmitate from acetyl-CoA and malonyl-CoA. Aberrant FASN function is associated with tumourigenesis in various human cancers, making it a potential cancer drug target [73–75]. CAV1 and FASN are coordinately regulated in melanoma and prostate cancers, implying that their concomitant expression may enhance tumourigenesis [76–78]. The mechanism explaining CAV1 and FASN interaction to promote tumourigenesis is still unknown, but a possible biochemical link is the dynamics in palmitoylation – a posttranslational modification process, in which long chain fatty acids (mainly palmitate) bind to cysteine residues in proteins via a thioesther bond. CAV1 has palmitoylated residues (Fig. 3a), and the effects of palmitoylation on membrane proteins include regulation of membrane interaction, stability, spatial organisation, and intracellular trafficking reviewed in [79, 80], all of which could alter how CAV1 interacts with metabolic targets. It is likely that by being co-expressed with FASN, CAV1 ensures availability of the lipids required for maintaining membrane integrity of tumour cells. Insights could be gained from study in visceral adipose tissue (VAT) of obese patients where a positive correlation between *CAV1* expression and lipogenic genes, e.g. acetyl-CoA carboxylase and *FASN* was reported [81].

CAV1 modulates several lipid metabolic processes in hepatocytes, including hepatic lipid storage, fatty acid oxidation (FAO) and ketogenesis [57, 58, 82]. For instance, *Cav1* deletion led to reduced hepatic triglyceride content and impaired lipid storage [58]. Furthermore, genomic profiling of liver, adipose tissue, and MEFs from fasted CAV1-deficient mice, all showed downregulation of lipid metabolic processes as a major consequence of loss of CAV1 [82]. These findings corroborate a previous report on reduced whole body FAO in *Cav1*-KO mice [57]. The reduction of FAO and ketogenesis in CAV1-deficient mice is attributed to impaired hepatic peroxisome proliferator activated receptor α (PPARα) activity [82]. Accordingly, although some PPARα target genes (e.g. *Gsta2*, *Scarb1*, *Nox4* and *Per2*) were upregulated in the CAV1-deficient mouse liver, there was a predominant suppression of several others, including carnitine palmitoyltransferases (*Cpt1α* and *Cpt1β*), acetyl-CoA acyltransferase 2 (*Acaa2*), and dehydrogenase/reductase (SDR Family) – *Dhrs4* and *Dhrs8*. In line, over 15 genes involved in peroxisomal and mitochondrial FAO, such as *Acox1*, *Hadhb*, *Cpt2* and *Acadm*, were downregulated upon loss of CAV1. Ketogenesis was likewise suppressed as confirmed by downregulation of 3-hydroxybutyrate dehydrogenase and reduced plasma level of β-hydroxybutyrate [82]. These findings underscore a strong link between CAV1 and lipid metabolism that is yet untested in cancer settings. Further evidence reveals that loss of CAV1 causes a switch from lipid towards glucose metabolism, as observed in endothelial cells [52], and murine hepatocytes [58]. Accordingly, decreased glycolytic intermediates after CAV1 knockdown in endothelial cells was attributed to accelerated utilisation, whereas increased level of fatty acids, such as palmitate, arachidonate, myristate, and essential fatty acids (e.g. linoleic and linolenic acids) were linked to a defective lipid metabolism [52]. Similarly, analysis of liver tissue showed that CAV1-deficient mice resorted to carbohydrate – instead of fatty acid metabolism [58]. Whether CAV1 influences such a crucial switch in cancer is not yet reported. Indeed, it is worthy to recall that lipids are important for raft remodeling – a crucial function of CAV1 [23, 25, 26]. Therefore, beyond modulating lipid metabolism, CAV1 depletion can compromise the functions of other molecules involved in lipid raft function. An example of such molecule is fatty acid-binding protein 7 (FABP7), which binds to and facilitates uptake of long-chain fatty acids. Recently, it was found that CAV1 mediates lipid raft activity of FABP7, namely receptor accumulation [83]. Specifically, knockout of *Fabp7* in mice suppressed CAV1 and ligand-dependent accumulation of Toll-like receptor 4 (TLR4) in astrocytes upon lipopolysaccharide stimulation, whereas overexpression of CAV1 in FABP7-deficient astrocytes was sufficient to restore TLR4 recruitment [83].

CAV1 level may determine the effect of lipid load on cancer cells. For instance, docosahexaenoic acid (DHA)– an omega-3 polyunsaturated fatty acid – is associated with cancer risk, progression and therapy [84, 85]. CAV1 participates in DHA mediated apoptosis in MDA-MB-231 cells [86]. Accordingly, DHA treatment caused cholesterol-dependent co-localisation of CAV1 and epidermal growth factor receptor (EGFR) with lysosome associated membrane protein 1 (LAMP-1), with an onward down-regulation of lipid-raft associated onco-proteins, including EGFR, HSP90, AKT and SRC [86]. Furthermore, expression of tyrosine-14 phosphorylated CAV1 accelerated palmitate-induced apoptotic cell death in pancreatic beta cells [87]. Taken together, these studies reveal that CAV1 is associated with fatty acid metabolism, offering insights for onward investigation in cancer. It will also be interesting to determine whether differential expression of CAV1 influences the expression or function of fatty acid enzymes that have recently emerged to be important mediators in cancer, e.g. acyl-CoA synthetase, carnitine palmitoyltransferase 1C, and carnitine acyltransferase [88–90].

## Caveolin-1 in metabolic abnormalities – obesity and insulin resistance

Obesity and insulin resistance, the major cause of type 2 diabetes (T2DM), are two intricately connected metabolic abnormalities of significant global health burden, and are strongly linked to cancer [91, 92]. For instance, in the case of obesity, there has emerged sufficient evidence in humans that reduced body fatness also reduces the risk of most cancers, prominent among which are liver, pancreatic, ovarian cancers, and multiple myeloma [92]. Similarly, a recent consensus report concluded that although several questions remain unanswered, “diabetes (primarily type 2) is associated with increased risk for some cancers (liver, pancreas, endometrium, colon and rectum, breast, bladder)” though also the reduced risk of prostate cancer [93]. Thus, whether in obesity and insulin resistance, understanding the activity of CAV1 could shed light on its role in metabolism that may be applicable in cancer. Indeed, studies in cancer and normal cells show that CAV1 modulates insulin signaling [48, 49, 57]. The loss of CAV1 leads to insulin resistance, and several of its genetic variants are associated with type 2 diabetes and lipid disorders [94]. It is believed that CAV1 stabilizes insulin receptor and so enhances cellular response to insulin stimuli as is necessary for promoting glucose uptake and metabolism. Besides, in a differentiating murine preadipocyte model 3T3-L1, *Cav1* promoter hypomethylation caused its overexpression and phosphorylation of its protein, accompanied by increased expression of insulin receptor, glucose transporter 4, and adipokines (adiponectin, interleukin 6 and leptin) [95]. CAV1 expression, therefore, apparently contribute to both insulin sensitivity and obesity. Consistent with the latter, CAV1 is overexpressed in VAT and subcutaneous adipose tissue of obese patients with normoglycaemia or T2DM (basically characterized by hyperglycemia and hyperinsulinemia) – and is positively correlated with body fat and body mass index [96]. Although still poorly understood, the mechanistic link between obesity, CAV1 and cancer may be partly due to increased adipokine activity. For instance, murine high fat diet model of obesity exposed to anti-obesity drug (orlistat) or restricted caloric intake had smaller adipocyte size, low CAV1 and suppressed adipokines (leptin and resistin) – in vivo growth of murine (B16F10) and human (A375) melanoma cell lines was also reduced in the obese mice [97]. Interestingly, the authors showed that stimulation with the adipokines enhanced proliferation and CAV1 level in A375 cells in vitro [97]. This finding supports a potential involvement of CAV1 via adipokines in obesity-driven cancer. Indeed, the involvement of CAV1 or its genetic variants in obesity and insulin resistance offers a pathophysiological clue for future understanding of its role in tumour glucose and fatty acid metabolism.

## CAV1 in autophagy

Autophagy is a process in which cells breakdown their cytoplasmic components to release metabolites for meeting nutritional needs [98]. Thus, autophagy is either a routine metabolic process in itself or is a pivotal mechanism initiated for long-term cellular energy homeostasis, especially during starvation [98, 99]. Autophagy is crucial in cancer, which explains why its inhibition leads to cancer cell death [100–102]. In addition, there is evidence that autophagy in surrounding tumour cells, as recently shown in pancreatic stellate cells [11], may actually be key for tumour nutrient supply in the hypovascularized microenvironment. Several studies have found autophagy to be very context-dependent. Thus, while CAV1 is implicated in the regulation of autophagy reviewed in [33, 103], it is not surprising that the molecular mechanisms seem inconsistent – supporting both direct and inverse associations. For example, in HCC, CAV1 inhibited autophagy while promoting cell proliferation, migration, and angiogenesis [104]. Accordingly, CAV1 inversely correlated with autophagy markers ATG5 and BECLIN-1 in clinical HCC and cell lines, while its knockdown increased autophagosome formation [104]. A well-acknowledged marker of autophagic activity commonly used to indicate increased autophagosome formation or decreased turnover is the microtubule-associated protein 1 light chain 3 beta (LC3B) [105, 106]. The suppression of CAV1 markedly increased LC3B-II in endothelial cells [52]. CAV1 deficiency in breast cancer also promoted late stage autophagy by enhancing lysosomal function and autophagosome-lysosome fusion towards improved cell survival under nutrient starvation [107]. Similarly, knockdown of CAV1 in stromal fibroblast induced autophagy, and hypoxia-induced degradation of CAV1 led to upregulation of autophagy markers [108].

Studies also show that CAV1 is directly associated with or regulates autophagy. For example, CAV1 correlates with estradiol-mediated autophagy in the BT474 breast cancer cell line. These cells expressed higher CAV1 and autophagy-related proteins (BECLIN-1, light chain (LC3)-II and ATG12/5) upon estradiol treatment [109]. In lung epithelial cells, CAV1 interacts with and regulates expression of autophagy proteins, notably ATG12, ATG5, and the ATG12-ATG5 complex that is important for autophagosome formation [110]. CAV1 is also an upstream regulator of transient receptor potential melastatin 3 (TRPM3), which controls Ca^2+^ and Zn^2+^ flux, and induces oncogenic autophagy via LC3A/B-II [111]. In this setting, TRPM3 expression requires CAV1, while knockdown of their encoding genes reduce LC3A/B-II accumulation in renal carcinoma cell lines [112]. *Cav1* was also among genes downregulated following bleomycin-induced pulmonary fibrosis in mice with autophagy gene *Atg4b* deletion [112]. The downregulation of *Cav1* coincided with altered expression of several profibrotic genes, including upregulation of *Tgfbr2*, *Smad3*, *Tgfb1*, *Tgfb3*, and suppression of *Smad4*, *7* and *Snai1* [112], suggesting a link between its expression and fibrosis via autophagy. In non-small lung adenocarcinoma cells (A549), expression of CAV1 was protective by blocking a switch from autophagy to apoptosis [113]. Taken together, these studies show a context-dependent involvement of CAV1 in autophagy that requires further clarification, especially in cancer types where CAV1 is a key player.

## Conclusions

There is abundant evidence that CAV1 plays diverse roles in normal and cancer cell metabolism (Figs. 1 and 2), which appears to be spatiotemporal and dependent on cell types, microenvironmental factors, nutritional availability and probably even additional perturbations, e.g. hypoxia or drug treatment. For instance, while CAV1 overexpression may drive glycolysis and glucose dependency, its knockdown also enhances glycolytic phenotypes in cancer and normal cells. Similar discrepancies also exist in autophagy, where different studies show that both loss and overexpression of CAV1 induces the lysosomal marker LC3B. Given these conflicting findings, more studies are required to unravel the exact mechanisms by which CAV1 controls cell metabolism – especially in cancer types associated with its high expression. Based on evidences from literature, we propose that mechanisms by which CAV1 modulate cancer metabolism may include: enabling the expression of membrane-localized metabolic enzymes; coordinating a switch between metabolic pathways; relaying signals for transcriptional activation or suppression of metabolic regulators, or inhibiting anti-metabolic stimuli (Fig. 3b).

To understand the role of CAV1 in cancer metabolism, studies should employ experimental models that are as close to the human cancer situation as is possible. In addition, studies designed to extend current knowledge on CAV1 function should endeavor to validate some previous observations using the same experimental models, i.e. cell and tissue types (Table 1). This will ensure consistency and also help to detect confounding experimental variables. Diseases such as obesity and diabetes could also provide an avenue through which molecular clues on the involvement of CAV1 in cellular metabolism can be unraveled for subsequent investigation in cancer. From a technical viewpoint, complementing CAV1 gain or loss of function studies with high throughput molecular biology methods, such as metabolomics, genomics and proteomics, will help to clarify its function in cancer cell metabolism.

**Table 1:** Cell, tissue types and disease conditions in which CAV1 was found to correlate with or influence metabolic processes

CAV1 promoted or directly correlated with metabolic processes	Ref.	CAV1 suppressed or inversely correlated with metabolic processes	Ref.
Glycolysis			
•Colon cancer [HCT116, HT29, LoVo]* •Prostate cancer [LNCaP, PC-3] Smooth muscle cells, lymphocytes	47, 48 49 51	BAEC CA fibroblast [hTERT–BJ1] Breast cancer cell [MCF7] Mouse liver (including gluconeogenesis)	52 55 56 58
Mitochondrial function			
BAEC Hepatic cells Microglia Colon cancer [HCT116, HT29] MEFs	52 59 60 61 57, 62	NIH3T3 cells oncogenically transformed with H-RAS mutant	43
Glutamine metabolism			
*Xenopus laevis* oocyte	70	Stromal fibroblast; [hTERT–BJ1]	64, 103
Fatty acid metabolism			
BAEC	52	–	–
Mouse liver	57, 58, 82		
Mouse hepatocyte [AML12]	58		
Adipocytes	57, 82		
MEFs	57, 82		
Melanoma, prostate cancer	76, 77, 78		
Metabolic diseases			
Obesity:		Diabetes:	
Adipose tissues of patients	96	Insulin insensitivity, hyperglycemia	57
Murine preadipocyte model [3T3-L1]	95	Insulin resistance (Type 2 diabetes)	94
High fat diet mouse model	97		
Autophagy			
Breast cancer cell [BT474] treated with estradiol	109	BAEC	52
Lung epithelial cells [BEAS-2B]	110	•Hepatocellular carcinoma [HCCLM3]*	104
•Renal carcinoma cell [786-O, A498]*	111	•Breast cancer [MCF7, MDA-MB-231]*	107
Murine fibrotic lung tissue	112	MEFs	107
Lung cancer cell [A549]	113	CA fibroblast [hTERT–BJ1]	108

MEFs mouse embryonic fibroblasts, BAEC Bovine aortic endothelial cells, CA Cancer associated; •Including in clinical samples; In squared bracket [] are cell lines used for mechanistic studies; *other cell lines are mentioned in the studies

Finally, the identification of key initiating factors – whether genomic or signaling mutations – that drive metabolic alterations are crucial steps in fine-tuning anti-cancer strategies. For instance, if low CAV1 expression in cancer also causes high dependency on glucose over lipid metabolism as reported in non-cancer cells [52, 58], then it raises an attractive prospect of using CAV1 expression levels to stratify cancer patients into those that could benefit from either inhibitors of glucose or lipid metabolism. Interestingly, we highlighted evidences that CAV1 interacts with known mediators of altered metabolism, e.g. LDHA, PKM2 and FASN, which are currently being studied as cancer drug targets [17, 114, 115]. It is therefore conceivable that understanding the expression and function of CAV1 in cancer could serve as a pointer for exposing novel metabolic genes or pathway alterations of clinical importance.

## Abbreviations

∆ψ: Mitochondrial membrane potential
ALDOs: Aldolases
ATP: Adenosine triphosphate
CAV1: Caveolin-1
ETC.: Electron transport chain
FASN: Fatty acid synthase gene
G6PD: Glucose-6-phospate dehydrogenase
GLUTs: Glucose transporters
HCC: Hepatocellular carcinoma
HIF: Hypoxia inducible factor
HK2: Hexokinase 2
IGF-1: Insulin-like growth factor 1
KO: Knockout
LDHA: LDHD, Lactate dehydrogenase A, D
MCTs: Monocarboxylates
MEFs: Mouse embryonic fibroblasts
OAA: Oxaloacetate
OXPHOS: Oxidative phosphorylation
PFK: Phosphofructokinase
PKM2: Pyruvate kinase, muscle isoform
PPP: Pentose phosphate pathway
SLCs: Solute carrier families
TCA: Tricarboxylic acid
VAT: Visceral adipose tissue
αKG: Alpha ketoglutarate
